# A dynamical model improves reconstruction of handwriting from multichannel electromyographic recordings

**DOI:** 10.3389/fnins.2015.00389

**Published:** 2015-10-29

**Authors:** Elizaveta Okorokova, Mikhail Lebedev, Michael Linderman, Alex Ossadtchi

**Affiliations:** ^1^Centre for Cognition and Decision Making, National Research University Higher School of EconomicsMoscow, Russia; ^2^Department of Neurobiology, Duke UniversityDurham, NC, USA; ^3^Department of Biomedical Engineering, Norconnect Inc.Ogdensburg, NY, USA; ^4^Laboratory of Control of Complex Systems, Institute of Problems of Mechanical Engineering, Russian Academy of SciencesSt. Petersburg, Russia

**Keywords:** handwriting, electromyography, pattern recognition, dynamical modeling, the Kalman Filter, the Wiener Filter

## Abstract

In recent years, several assistive devices have been proposed to reconstruct arm and hand movements from electromyographic (EMG) activity. Although simple to implement and potentially useful to augment many functions, such myoelectric devices still need improvement before they become practical. Here we considered the problem of reconstruction of handwriting from multichannel EMG activity. Previously, linear regression methods (e.g., the Wiener filter) have been utilized for this purpose with some success. To improve reconstruction accuracy, we implemented the Kalman filter, which allows to fuse two information sources: the physical characteristics of handwriting and the activity of the leading hand muscles, registered by the EMG. Applying the Kalman filter, we were able to convert eight channels of EMG activity recorded from the forearm and the hand muscles into smooth reconstructions of handwritten traces. The filter operates in a causal manner and acts as a true predictor utilizing the EMGs from the past only, which makes the approach suitable for real-time operations. Our algorithm is appropriate for clinical neuroprosthetic applications and computer peripherals. Moreover, it is applicable to a broader class of tasks where predictive myoelectric control is needed.

## 1. Introduction

Handwriting is a unique development of human culture. A skill learned during the early childhood, it remains among the primary means of communication and self-expression throughout the course of life. From the physiological point of view, handwriting is a complex interplay between the nervous system and the numerous muscles of the upper extremity. Despite several attempts to study this intricate activity theoretically (Plamondon and Maarse, 1989; McKeague, 2005) and experimentally (Linderman et al., 2009; Huang et al., 2010; Li et al., 2013), it is still not well understood and can not be reliably replicated in prostheses.

The relationship between the muscle force and the pen trajectory is complicated by the motor redundancy phenomenon (Bernstein, 1967; Guigon et al., 2006). One and the same movement can be accomplished via basically infinite number of muscle activation patterns. Relatively fine spatial scale inherent to the handwriting process and the natural variations of the limb's kinematic variables further complicate the issue.

Additional obstacle in studying the physiology of handwriting is the difficulty of measuring the muscle force directly. Surface and intramuscular Electromyography (EMG) are the common methods to register neuromuscular activity during a motor task. Surface EMG is a non-invasive method which implies placing the electrodes on the skin above the muscles of interest. Being easy and safe to implement, this technique, however, does not yield sufficiently accurate biomechanical measurements, due to the complex relationship between the EMG and muscle force, complicated anatomy of muscles and the inability to record from all the muscles involved, especially from the deep muscles. Intramuscular EMG (iEMG) is invasive and uses needle electrodes inserted into the muscle tissue to yield more spatially specific measurements with less leakage and disturbance. The invasive nature of iEMG limits its utility.

Both EMG registration methods posit several substantial difficulties, primarily related to signal quality and associated issues of noise filtering and source extraction from the observed data. Nonetheless, it was shown that even the surface EMG carries valuable information about the neuromuscular interactions and can therefore be used effectively in modeling and interpreting movements (Reaz et al., 2006; Ahsan et al., 2009).

Despite the evident difficulties of measuring and interpreting neuromuscular activity with the currently available techniques, understanding such complex motor tasks as handwriting is important for both theoretical and practical reasons. Once we learn how to model the relationship between EMG patterns and pen movements during handwriting, we can introduce this knowledge to many rapidly expanding fields and practices, including biomedical engineering, robotics and biofeedback therapy. For instance, we can substantially improve the existing treatment and rehabilitation techniques for patients with a loss or an injury of an upper limb (Xiao and Menon, 2014), create rules for diagnostics of motor diseases based on handwriting (Van Gemmert et al., 1999; Stanford, 2004; Silveri et al., 2007), and even assist young children in learning how to write (Carter and Russell, 1985). Besides, an accurate model and methodolgies for building such models, establishing the correspondence between the handwriting and muscle activation patterns has a potential to become a foundation for creating intelligent neural prosthesis with a substantial number of degrees of freedom and fine spatial scale (Chan et al., 2000; Ohnishi et al., 2007; Shenoy et al., 2008; Bu et al., 2009; Castellini and van der Smagt, 2009).

However, such appealing advances and practices are still in their infancy. To date, the existing research on decoding of handwriting from electromyography is small and restricted to laboratory conditions. Several papers addressed the question of written character classification based on surface EMG, which involved implementation of machine-learning techniques to distinguish between muscle activation patterns for different written characters, such as digits, alphabet letters or simple geometric shapes. Linderman et al. (2009) classified symbols from 0 to 9, using eight bipolar surface electrodes placed on the hand and the forearm muscles. They implemented Fisher Linear Discriminant Analysis to obtain, on average, 90% accuracy of classification across subjects. Huang et al. (2010) used Dynamic Time Warping (DTW) to classify symbols based on 6-channel EMG recordings. Their average classification accuracy was 98.25% for digits, 97.89% for Chinese symbols and 84.29% for Latin capital letters. Li et al. (2013) improved the DTW algorithm by substituting Euclidean Distance with Mahalanobis Distance, to increase classification precision to almost 95%. In their experiment, subjects were instructed to write lower-case letters, while 4-channel EMG signals were recorded from their forearm.

The other studies considered a rather complex task of on-line decoding of the pen traces, based on the incoming EMG signals from the measurement electrodes. Among the most successful methods known to the authors, is the Wiener Filter (Linderman et al., 2009), which allows to attain accuracy of reconstruction of 47 ± 2% for X-coordinate and 63 ± 15% for Y-coordinate, measured by the coefficient of determination. However, the method used the data samples from the future, which would lead to extra delays in cases when used in the on-line mode.

In this paper, we consider the same multisubject data-set as in Linderman et al. (2009) and present our approach to EMG-based pen tracking that by taking into account the dynamic model of the pen coordinate process allows to outperform the previously reported techniques. The main idea behind our method is to fuse two information sources available about the process of handwriting. The first information source comes from the physical and the kinematic characteristics of handwriting. The second information source comes from the multichannel electromyography that indirectly measures the strength of the upper extremity muscles, activated to move the pen. To perform the fusion of the two sources optimally, we employ the Linear Kalman Filter (Kalman, 1960), which is a well-known recursive algorithm for dynamic statistical model-based inference.

## 2. Materials and methods

### 2.1. The Kalman Filter

#### 2.1.1. Preliminary remarks

In its classical formulation the Kalman Filter (KF) (Kalman, 1960) is an algorithm that fuses several (usually two) noisy sources of information to produce an estimate of the dynamical system's state vector, which is optimal in the “minimum squared error” sense. The method is over 50 years old, but it is still very popular, due to its intuitive structure, ease of implementation and computational efficiency.

In our application, the first information source is the dynamical model that captures the physical properties of the arm-wrist-pen device and is formalized as a multivariate autoregressive (MVAR) process, whose parameters are estimated from the data. The noisy vector of EMG measurements is the second source of information, whose relation to the pen coordinate is modeled via multivariate linear regression equation with coefficients determined from the training data-set.

For simplicity, the derivations provided in this section are based on the assumption of multivariate normality of the fused sources (Faragher, 2012), which is essential for the Kalman Filter to be the optimal estimator (the best among all other kinds). However, in the general Kalman Filter framework, this does not have to be the case (Arulampalam et al., 2002). When the assumption of normality does not hold, it is still possible to derive the KF equations based on the orthogonality principle (e.g., Jazwinski, 2007), which would guarantee the KF to be the best linear estimator, but not necessarily be optimal. In this case, it might be possible to increase accuracy by employing non-linear techniques that exploit higher order dependencies in the data, such as the Extended Kalman Filter (Julier and Uhlmann, 1997) or its “distribution-free” version, called Unscented Kalman Filter (Wan and Van Der Merwe, 2000). However, since the majority of trials in our multisubject dataset appear to test positively for the normality (see Appendix, Section Testing the Assumptions of the Model) the extent of improvement furnished by the use of the non-linear approaches is hard to predict theoretically. This leaves the question in the empirical realm to be addressed in the future studies.

As demonstrated by the statistical tests described in the Appendix (Section Testing the Assumptions of the Model), we could not reject the hypothesis of independence for the majority of trials. Based on this and, for the simplicity reasons, we base our developments in this paper on the assumption of independence of the two fused sources. In case the independence assumption is violated, the performance gained by taking into account the dynamics of the reconstructed process could have been more sizable should we use a slightly modified form of equations (Shimkin, 2009) to account for the non-trivial cross-covariance structure of the residuals. However, the extent to which modeling the cross-covariance structure of residuals would improve the performance is not entirely clear, due to the inherent non-stationarity and the associated estimation errors.

#### 2.1.2. State transition model

As the first information source, we assume that, at each time moment *t*, the system evolves from the previous state at time *t* − 1, according to the rule:

(1) $$s_{t}=As_{t-1}+v_{t}$$

where

- $s_{t}=[x_{t},y_{t},{\dot{x}}_{t},{\dot{y}}_{t},{\ddot{x}}_{t},{\ddot{y}}_{t},...,x_{t-K+1},y_{t-K+1},{\dot{x}}_{t-K+1},{\dot{y}}_{t-K+1}$, ${\ddot{x}}_{t-K+1},{\ddot{y}}_{t-K}{]}^{T}$ is a 6*K* × 1 state vector containing pen coordinates and their first and second rates of change for the window of *K* time moments starting from *t*;
- ***A*** is a [6*K* × 6*K*] state transition matrix, which performs the mapping between the state vectors at the two consecutive time moments;
- ***v***_*t*_ is a [6*K* × 1] vector containing process noise, which is assumed to be drawn from a multivariate Gaussian distribution with zero mean and covariance matrix ***Q***.

Based on Equation (1), we can derive the following expressions connecting the mean and the covariance matrix of the state vector at the two consecutive time moments.

- **μ**_1*t*_ = ***Aμ***_1(*t*−1)_ is a 6K-dimensional mean state vector at time *t*;
- $\Sigma_{1t}={A\Sigma }_{1\left(t-1\right)}A^{T}+Q$ is a 6*K* × 6*K* positive-definite covariance matrix of the state vector at time *t*.

Detailed derivations of the model parameters can be found in the Appendix (Section Testing the Assumptions of the Model).

#### 2.1.3. Measurement model

Usually, in the KF framework, the measurement equation appears in the ***z*** = *F*(***s***) form, describing the way the process to be estimated (***s***) is related to the available vector of indirect measurements (***z***). However, in our application, due to causal and physiological reasons, it is more natural to think that the EMG registered muscle activity gives rise to the pen movement. Therefore, we use the “inverse” form of what is usually called the observation equation in the KF framework and write

(2) $$s_{t}=Hz_{t}+w_{t}$$

where

- ***z***_*t*_ is a [8*L* × 1] observation vector containing *L* groups of eight EMG measurements corresponding to the [*t* − *L* + 1, *t*] window of *L* most recent samples;
- ***H*** is a [6*K* × 8*L*] measurement transformation matrix, mapping the measurement domain to the state vector domain;
- ***w***_*t*_ is a [8*L* × 1] vector of measurement noise with zero mean and covariance matrix ***R***. Additionally, the measurement noise ***w***_*t*_ is assumed to be independent from the process noise ***v***_*t*_.

The 6*K*-dimensional state mean vector at time *t* is given by

(3) $$\mu_{2t}=E[s_{t}]=Hz_{t}.$$

Since we do not model ***z***_*t*_ as a stochastic process, the covariance matrix of *s*_*t*_ reduces to covariance matrix of the measurement noise, so that $\Sigma_{2t}=E\left[w_{t}w_{t}^{T}\right]=R$. Note that this noise is assumed to be stationary.

#### 2.1.4. Information fusion

**Figure 1:**
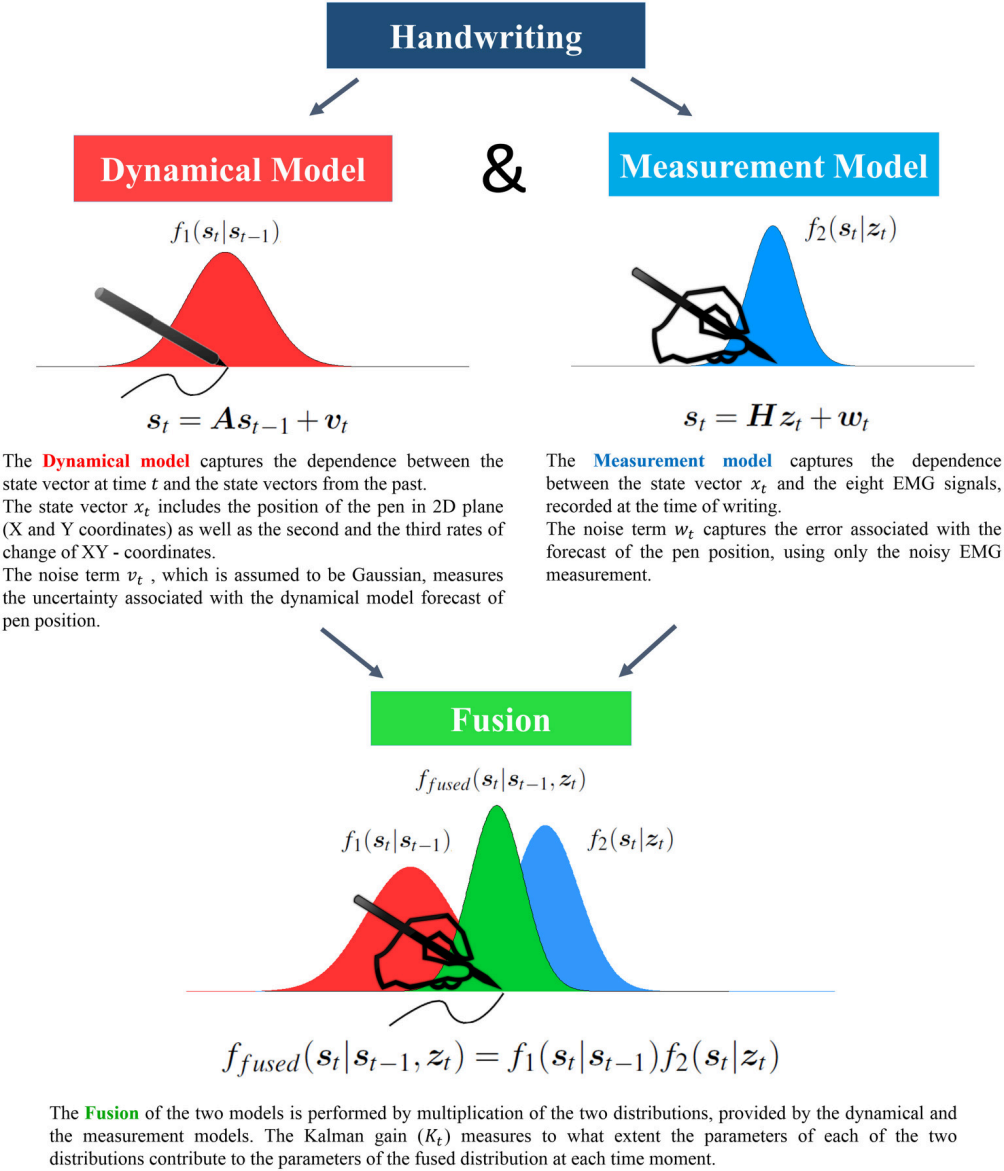
**Information Fusion by means of the Kalman Filter allows to improve reconstruction accuracy by adaptively balancing the contribution from the two information sources**.

As outlined in the previous two subsections, we have two independent sources of information about the state vector. The first endogenous source bases its predictions on the dynamical characteristics of the pen coordinates during the handwriting and yields *f*_1_(***s***_*t*_|***s***_*t*−1_) as the state vector distribution (red distribution in Figure 1). The second source is exogenous and uses externally registered EMG signals to suggest *f*_2_(***s***_*t*_|***z***_***t***_) as the state vector distribution (blue distribution in Figure 1). In order to reconstruct the state vector, optimally taking into account the predictions from both sources, we perform the statistical fusion of the estimates based on the dynamical and the measurement models. The schematic procedure of the source fusion is illustrated in Figure 1.

The joint conditional estimate of the state vector is distributed as *f*_*fused*_(***s***_*t*_|***s***_*t*−1_, ***z***_*t*_) (green distribution in Figure 1). Assuming independence of the sources, the problem of finding *f*(***s***_*t*_|***s***_*t*−1_, ***z***_*t*_) reduces to a simple multiplication of the two probability density functions, i.e.,

(4) $$f_{fused}(s_{t}|s_{t-1},z_{t})=f_{1}(s_{t}|s_{t-1})f_{2}(s_{t}|z_{t})$$

The product of two multivariate normal distributions is also a multivariate normal (Rencher, 2003). Its mean and covariance can be easily expressed in terms of the mean vectors and the covariance matrices of each of the two normal multipliers.

Specifically, **Σ**_*fused*_ is the covariance matrix of the fused distribution can be computed as

(5) $$\Sigma_{fused}={\left(\Sigma_{1t}^{-1}+\Sigma_{2t}^{-1}\right)}^{-1},$$

and the 6*K*-dimensional mean vector of the fused distribution is found to be the following weighted sum of the two mean vectors of the fused information sources:

(6) $$\mu_{fused}=\Sigma_{fused}(\Sigma_{1t}^{-1}\mu_{1t}+\Sigma_{2t}^{-1}\mu_{2t}),$$

It is instructive to reformulate the expressions for the mean and the covariance matrix of the new distribution and to separate the influence of the two distributions being fused.

Using the matrix inversion lemma (Henderson and Searle, 1981), and setting

(7) $$K_{t}=\Sigma_{1t}{\left(\Sigma_{1t}+\Sigma_{2t}\right)}^{-1}$$

we can rewrite Equations (5) and (6) as

(8) $$\Sigma_{fused}=\Sigma_{1t}-K_{t}\Sigma_{1t}.$$

and

(9) $$\mu_{fused}=(I-K_{t})\mu_{1t}+K_{t}\mu_{2t}.$$

For detailed derivation of the parameters see the Appendix.

The term ***K***_*t*_ in Equation (7), commonly known as the Kalman Gain, plays a crucial role of the dynamic scaling factor reflecting the distribution of trust in each of the two information sources. It depends on the relative amount of uncertainty present in the estimates by each of the information sources alone, and varies over time.

Since covariance matrices are positive definite, Equation (8) shows that by fusing the two distributions we reduce the variation associated with the state estimate, proportionally to the Kalman gain. At the same time, the fused mean (Equation 9) becomes the weighted average of the endogenously predicted and the measurements-based mean estimates.

#### 2.1.5. The algorithm

Based on the above equations we are now ready to formulate the algorithm for calculating the Kalman Filter estimate. At each time moment the computation can be split into three consecutive steps.

1. Endogenous state prediction and error covariance update:
  (10) $${\hat{s}}_{t|t-1}=\mu_{1t}=A{\hat{s}}_{t-1|t-1}$$
  (11) $$P_{t|t-1}=\Sigma_{1t}=AP_{t-1|t-1}A^{T}+Q$$
2. Kalman Gain Calculation:
  (12) $$K_{t}=\Sigma_{1t}{\left(\Sigma_{1t}+\Sigma_{2t}\right)}^{-1}=P_{t|t-1}{\left(P_{t|t-1}+R\right)}^{-1}$$
3. Measurement Update:
  (13) $${\hat{s}}_{t|z}=\mu_{2t}=Hz_{t}$$
  (14) $${\hat{s}}_{t|t}={\hat{s}}_{t|t-1}+K_{t}({\hat{s}}_{t|z}-{\hat{s}}_{t|t-1})$$
  (15) $$P_{t|t}=P_{t|t-1}-K_{t}P_{t|t-1}$$

In order to relate our approach to the classical KF paradigm in the equations above, we assigned the variables used in the previous subsection to the standard symbols, commonly employed in the KF literature. In the above algorithm, the first step is to use the State Transition Equation only and to calculate the so-called *a priori* estimate ${\hat{s}}_{t|t-1}$, with associated variance ***P***_*t*|*t*−1_ (Equations 10 and 11).

Then, we calculate the Kalman Gain based on the *a priori* covariance matrix and the covariance matrix of the Measurement Model (Equation 12).

Finally, the *a posteriori* estimate ${\hat{s}}_{t|t}$ is computed by adjusting the endogenous *a priori* estimate with the EMG measurements. The amount of adjustment is governed by the time-varying Kalman Gain (Equation 14) and the innovations process ${\hat{s}}_{t|z}-{\hat{s}}_{t|t-1}$, informing the algorithm on the amount of mismatch between the endogenous and exogenous estimates. The *a posteriori* uncertainty, associated with the prediction, based on the two models, is given by ***P***_*t*|*t*_ (Equation 15), which shows that, in the final estimate, the *a priori* uncertainty gets reduced proportionally to the Kalman Gain.

### 2.2. The experiment

#### 2.2.1. Data

Six healthy participants were instructed to write symbols from 0 to 9, repeating each symbol approximately 50 times. At the same time, muscle activity was recorded with eight bipolar-surface EMG electrodes, placed on each participants leading hand muscles: *opponens pollicis, abductor pollicis brevis, medial and lateral heads of first dorsal interrosseus*, and four forearm muscles: *flexor carpi radialis, extensor digitorum, extensor carpi ulnaris*, and *extensor carpi radialis*. The reference electrode was placed on each subject's forehead. Position of the pen was recorded using the special digitizing tablet, yielding a pair of coordinates in the two-dimensional space. For a detailed illustration of the experiment set-up, see Linderman et al. (2009).

#### 2.2.2. Preprocessing

Before applying the algorithm, we preprocessed EMG signals to extract the envelope via the standard rectification procedure. For each channel separately, we first calculated the absolute value of the EMG signals and then low-pass filtered the result with a second-order Butterworth Filter with the cut-off frequency of *F*_*c*_. We optimized the value of the cut-off frequency based on the training subset of the recorded data to obtain the best reconstruction performance. In the final results reported here *F*_*c*_ = 2 Hz. Additionally, we have applied square-root transformation to each signal's envelope, obtained via the described rectification procedure.

#### 2.2.3. Training and testing

**Figure 2:**
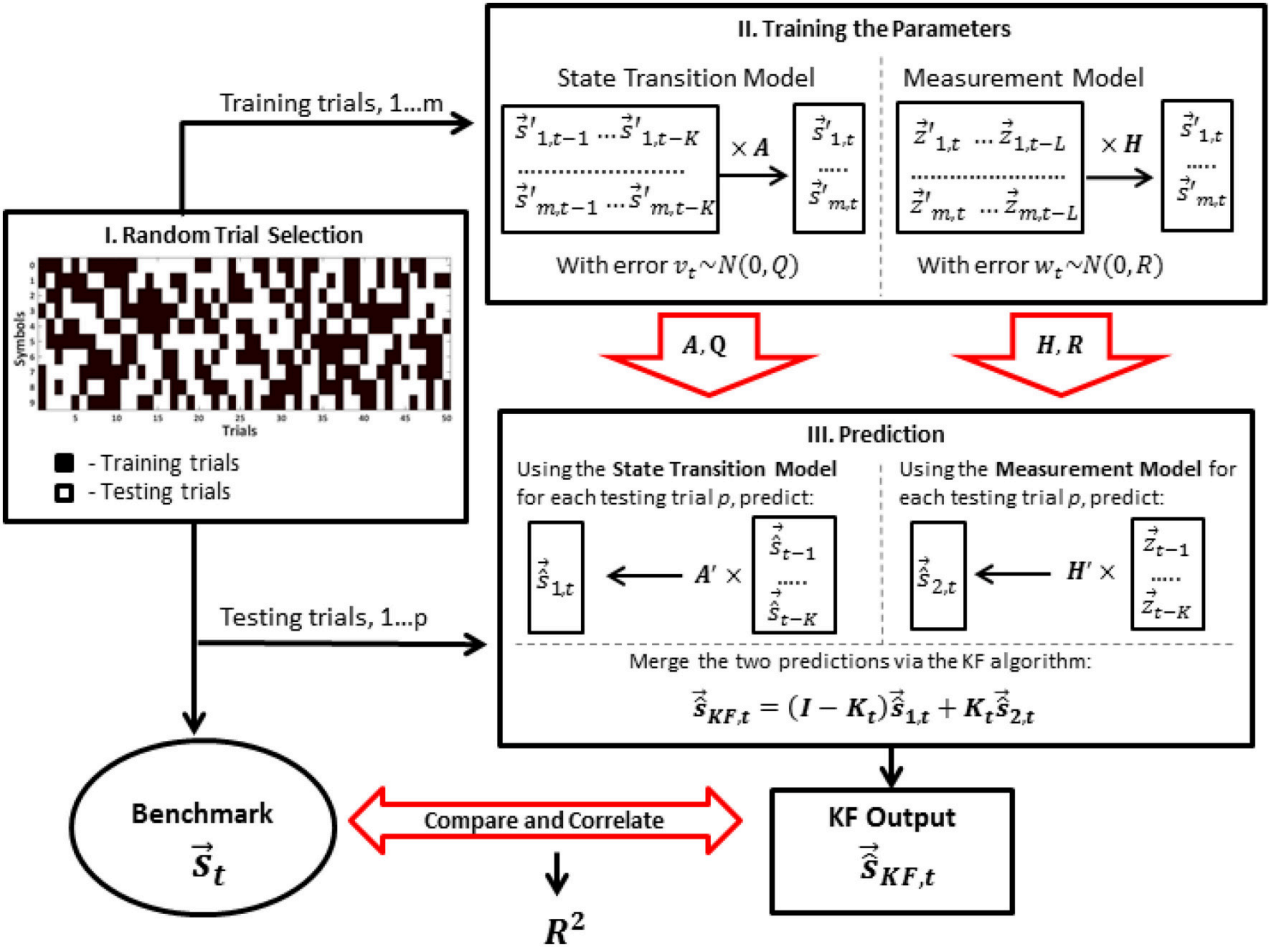
**Implementation of the Kalman Filter algorithm**. For each participant of the experiment, the trials are randomly divided into training trials (black) and testing trials (white). Then, the training trials are used for learning of the parameters of the state transition model (matrices *A* and *Q*) and the measurement model (matrices *H* and *R*). The EMG data from the testing trials and the learned matrices are then used for the prediction of the state vector in both models. Finally, the predictions of the two models are merged via the Kalman filter algorithm and the result of the filter is compared to the actual state vector. The squared correlation coefficient (*R*^2^) is used as a measure of efficiency of reconstruction.

Half of the trials of each symbol was randomly assigned to training the parameters of the model, while the remaining half was used for testing the performance (Figure 2). During training, the parameters of the dynamical model (Equation 1) and the measurement model (Equation 2) were estimated. We applied Ordinary Least Squares Method to estimate matrix ***A*** in the state transition equation and matrix ***H*** in the measurement equation. Covariance matrices ***R*** and ***Q*** were estimated based on the residuals of the two fitted models. Note that estimation of the covariance matrices of the error processes is particularly simple here, since, at the model identification step, we have the direct access to both state vector and the actual EMG measurements, thanks to the experimental setup described in Linderman et al. (2009). Figure 2 shows the data flow diagram in the model identification and coordinate reconstruction modes.

We used two basic experimental designs to calibrate our pen tracking algorithm.

1. Within-Group Design
  A single set of parameters (***A***, ***H***, ***R***, and ***Q***) was estimated using the training trials from all symbols at the same time and then tested on the remaining test trials.
2. Between-Group Design
  A separate set of parameters (***A***^*n*^, ***H***^*n*^, ***R***^***n***^ and ***Q***^***n***^, *n* ∈ 0, ..9) was estimated for each symbol and then tested within the data from the trials of the same symbol.

Note that for Within-Group Design, only one set of matrices was estimated by pooling all the training samples together, while in Between-Group Design the four matrices were estimated separately for each of the ten symbols. Then, the out-of-training sample measurements were used to reconstruct handwriting via the recursive process outlined in Section 2.1.5.

The testing procedure was the same within each experimental design (see Figure 2). For each trial, the starting pen location point was set to zero vector. Then, the estimate of the pen position was computed recursively (Section 2.1.5). Reconstruction accuracy was measured by the squared correlation coefficient ***R***^2^ between the actual coordinate and its fused estimate (Figure 2). This criterion corresponds to the percentage of energy in the actual pen traces (Total Sum of Squares - ***SS***_*t*_) explained by the reconstructed ones (Explained Sum of Squares ***SS***_*e*_), i.e.,

(16) $$R^{2}=\frac{SS_{e}}{SS_{t}}=\frac{\sum_{i\;=\;1}^{N}{\left({\hat{s}}_{i}-\bar{s}\right)}^{2}}{\sum_{i\;=\;1}^{N}{\left(s_{i}-\bar{s}\right)}^{2}}$$

The accuracy was computed within each trial, and then averaged across trials for each symbol. Confidence intervals were computed to account for the standard errors associated with the variation across the participants.

#### 2.2.4. Comparison with other models

We compared the accuracy of our model to the accuracy of the Wiener Filter (WF) estimate, which was originally tested on the same data-set by Linderman et al. (2009). The Wiener Filter approach is directly equivalent to predicting the pen coordinates, using only the measurement equation of the KF-framework alone (Equation 2). In other words, the Kalman filter is the Wiener filter, accompanied by information about the system's physical properties. Therefore, a comparison between the two models would not only show the possible improvement associated with the Kalman Filter, but also highlight the isolated benefit furnished by employing the dynamical properties of the system.

In the framework of handwriting recognition from electromyography reported in Linderman et al. (2009), the pen trace at time *t* was represented as a linear combination of EMG signals recorded in the non-causal interval of *t*: [*t* − δ_1_, *t* + δ_2_]. The unknown weights, mapping rectified EMG signals into the pen-tip coordinate vector, were estimated using the Ordinary Least Squares Method. It is important to stress that, in contrast to the approach reported in Linderman et al. (2009), our reconstruction procedures (both Kalman Filter and Wiener Filter based) operate causally and use only the samples from the immediate past. For each time moment *t*, only the samples from the [*t* − δ_1_, *t*] interval were used. It is, therefore, interesting and instructive to test, whether or not the use of the dynamical model compensates for the reduced amount of information in the external measurements. In the situation when both methods use the same amount of exogenously registered data, the Kalman Filter is expected to outperform the Wiener Filter. To test the hypothesis, we performed the coordinate reconstruction with the two filters, fixing all other external parameters related to the data preprocessing step and compared their performance on the testing set of trials.

## 3. Results

### 3.1. Finding optimal model order

**Figure 3:**
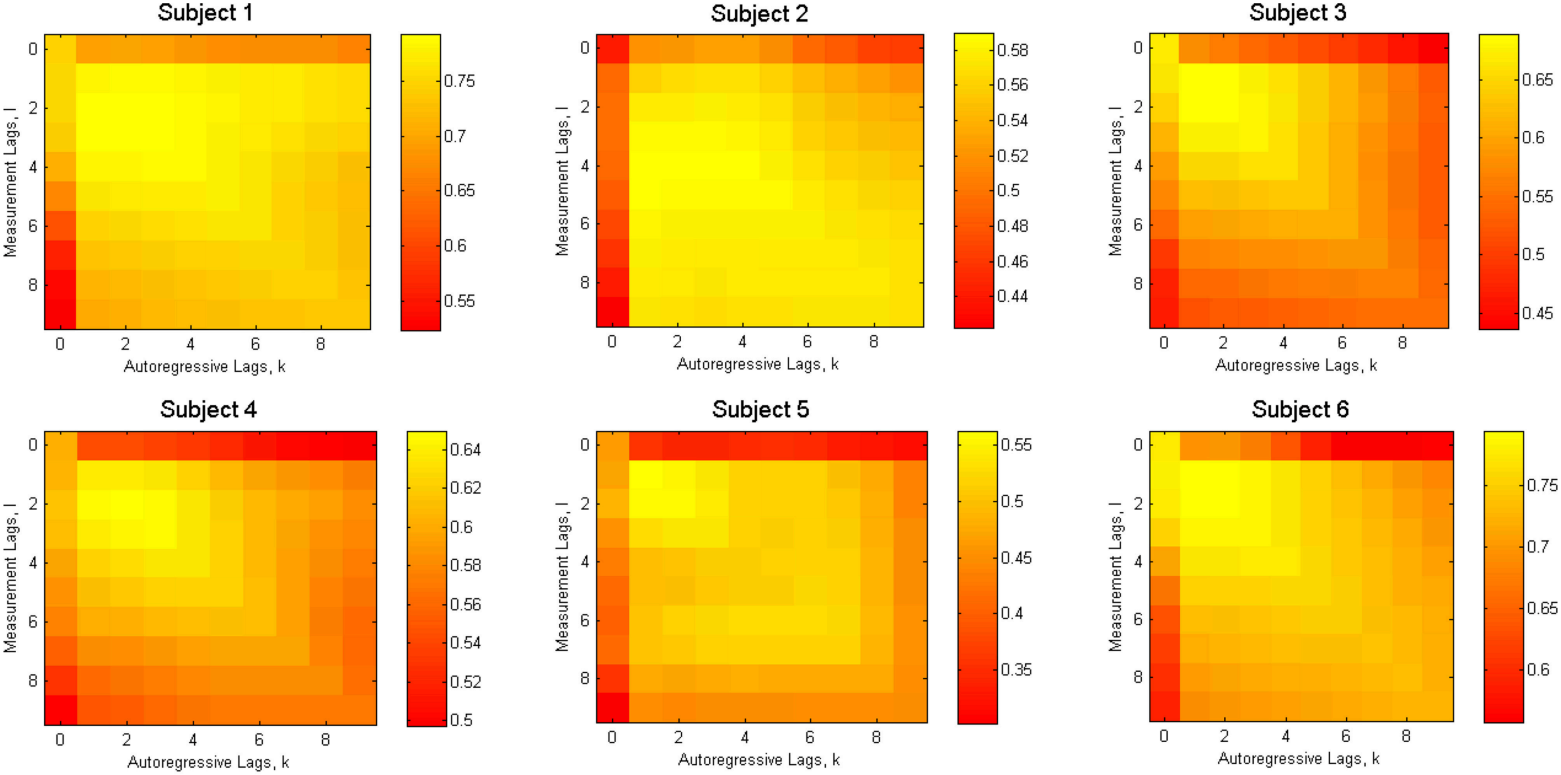
**Reconstruction accuracy as a function of model order parameters (*K* and *L*) for 6 subjects separately**.

The dynamical model (Equation 1) and the measurement model (Equation 2) contain model order parameters *K* and *L*, corresponding to the number of past samples used. In order to find the optimal values of these parameters, we applied the cross-validation procedure. We used ***R***^2^ as a metric of the goodness of reconstruction achieved (Figure 3).

**Figure 4:**
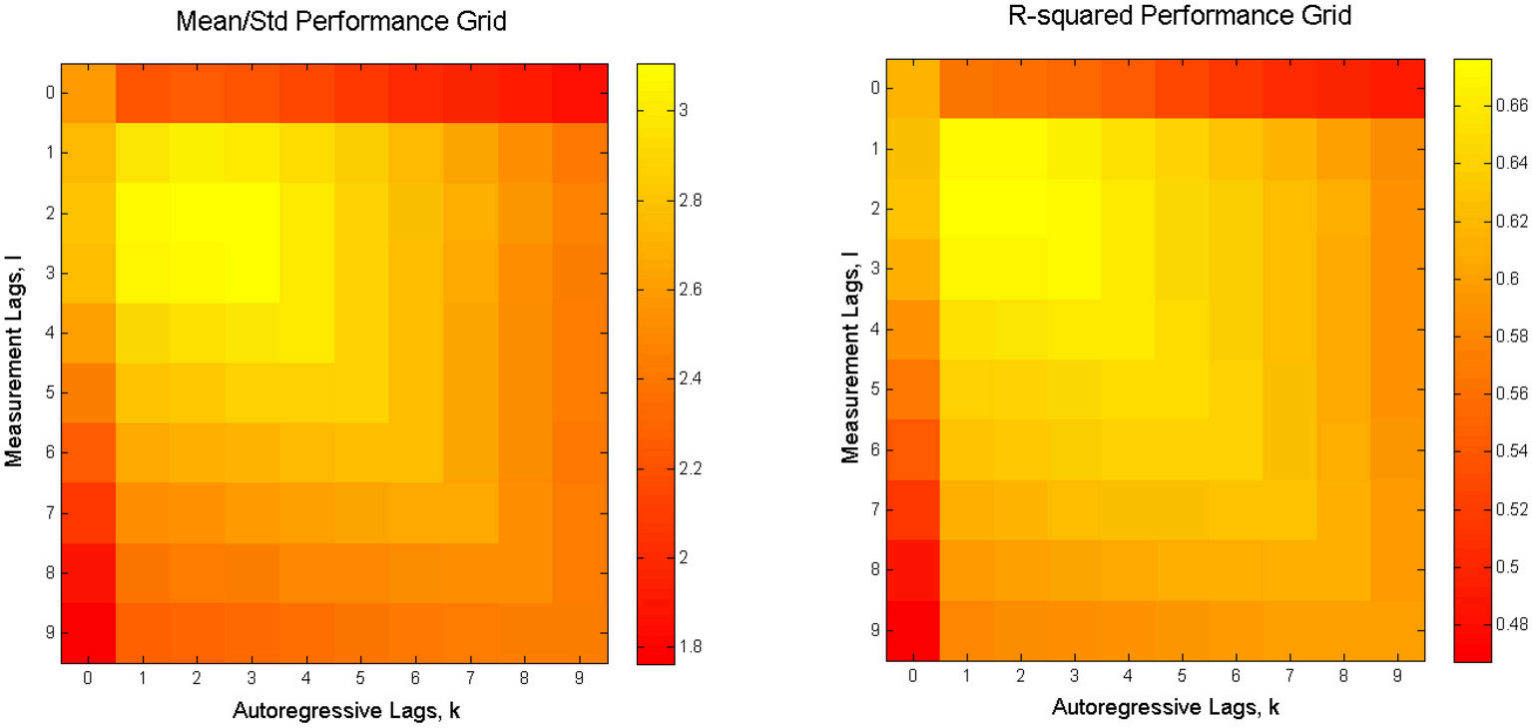
**Average reconstruction accuracy as a function of model order parameters (K and L)**. **Left:** average over all 6 subjects value of *g*. **Right:** Average over all 6 subjects value of *R*^2^ for the mean reconstruction accuracy in the two coordinates (*X*+*Y*)∕∕2.

To search for the combination of *K* and *L* that delivers the highest performance, we looked for the values of these parameters that maximize the *g* = *E*(***R***^2^)∕*std*(***R***^2^) ratio. The expected value *E*(***R***^2^) and the standard deviation *std*(***R***^2^) were computed over the trials in the data-set used for cross-validation. Therefore, high values of *g* correspond to the combination of high accuracy and stability of the reconstruction quality. Figure 4 shows the average over all subjects value of *g* and ***R***^2^.

Based on these maps, we set *K* = 1 and *L* = 2 to obtain the results reported in this paper. Note that the number of optimal measurement lags was reduced from 20 to 2, comparing to the original paper by Linderman et al. (2009), which significantly reduces computational complexity of the problem and reduces the response time of the system and potentially allows to track brisker movements.

### 3.2. Within-group design

**Figure 5:**
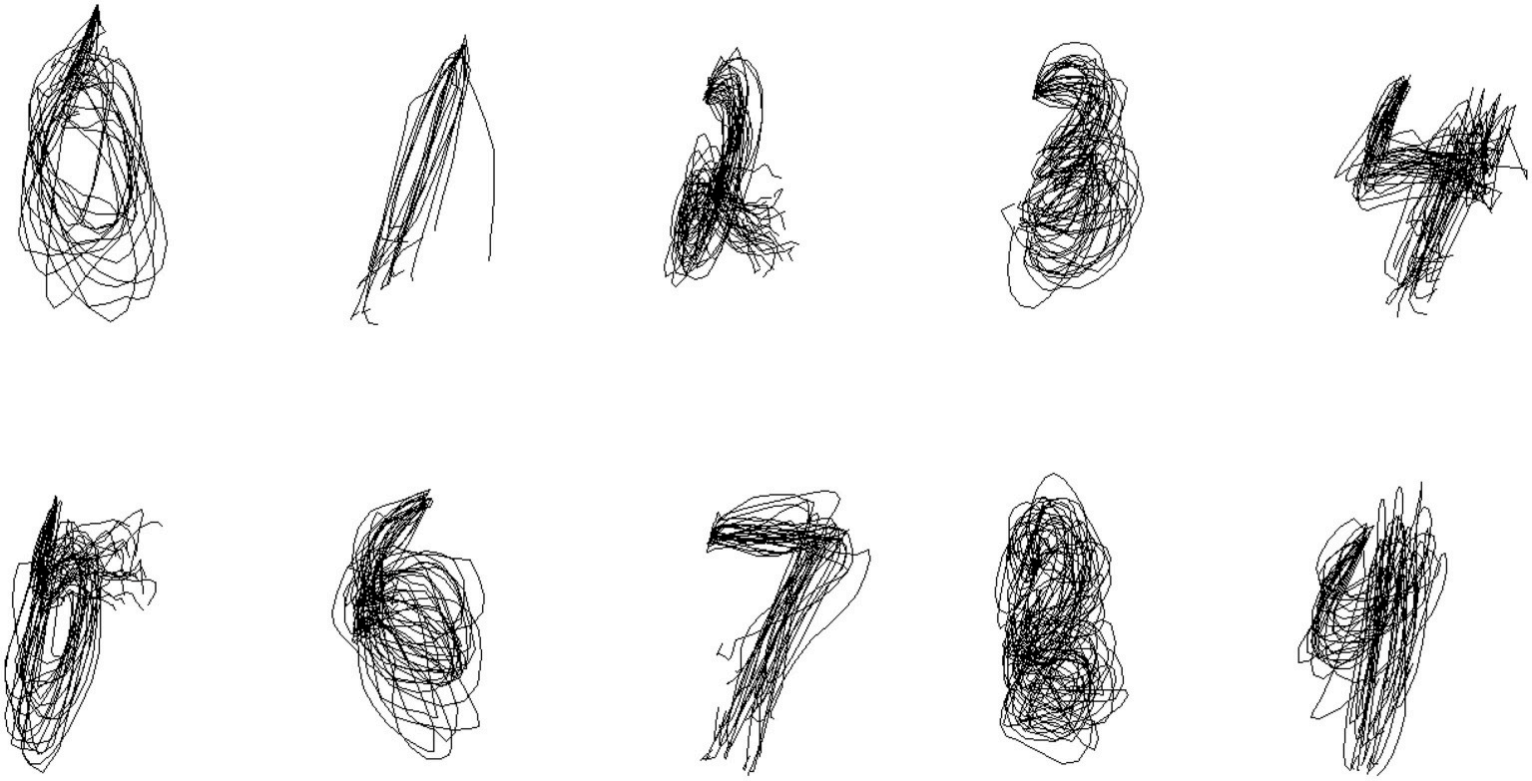
**Within-Group Reconstruction: several reconstructed trials of each symbol from cross-validation sample of one of the participants**.

We first trained one set of parameters for all symbols and used it to reconstruct pen traces from the EMG data in the testing set. Figure 5 shows the result of reconstruction of several trials of each character for one of the participants. Despite being noisy and, at times, inaccurate, the symbols are still identifiable and reproducible between trials.

Table 1 gives a detailed accuracy distribution for all symbols. Each of the entries in the table were found as follows: we computed accuracy of reconstruction for each test trial available for each symbol, then calculated the statistics within trial of the same symbol to determine the reported mean and standard deviation. Finally, we computed 95% confidence intervals for the average accuracy of every symbol reconstruction, based on the sample of 6 participants. We report reconstruction accuracy by coordinates independently, and as an average between the two coordinates.

**Table 1:** **Within-Group Reconstruction Performance: 95% confidence intervals for the average reconstruction accuracy of each symbol between the 6 participants**.

	**Average performance, *R*^2^**		
**Symbol**	**X-coordinate**	**Y-coordinate**	**Average: (X+Y)/2**
“0”	0.65 ± 0.15	0.57 ± 0.17	0.61 ± 0.15
“1”	0.49 ± 0.08	0.85 ± 0.08	0.67 ± 0.05
“2”	0.71 ± 0.14	0.75 ± 0.12	0.73 ± 0.13
“3”	0.59 ± 0.20	0.79 ± 0.09	0.69 ± 0.13
“4”	0.71 ± 0.15	0.63 ± 0.17	0.67 ± 0.13
“5”	0.67 ± 0.15	0.72 ± 0.05	0.70 ± 0.08
“6”	0.68 ± 0.18	0.71 ± 0.11	0.70 ± 0.12
“7”	0.59 ± 0.28	0.69 ± 0.22	0.63 ± 0.25
“8”	0.61 ± 0.15	0.74 ± 0.12	0.77 ± 0.11
“9”	0.62 ± 0.22	0.71 ± 0.15	0.67 ± 0.17
All	0.63 ± 0.17	0.73 ± 0.14	0.68 ± 0.13

Statistically speaking, we managed to achieve the average accuracy of 63 ± 17% and 73 ± 14% with 95% confidence, for the two reconstructed coordinates, as estimated for the six participants of the experiment. Note that the average accuracy in both coordinates is higher than that found by Linderman et al. (2009) (47 ± 2% and 63 ± 15% for the two coordinates, respectively), where non-causal Wiener Filter based reconstruction was employed. Additional improvement over that pioneering work lies in the use of the smaller number of measurement lags (L) (2 instead of 20, see Section 3.1) which reduces the response time of the system. These observations demonstrate the benefits brought in by the use of the dynamical properties of the process being identified.

### 3.3. Between-group design

**Figure 6:**
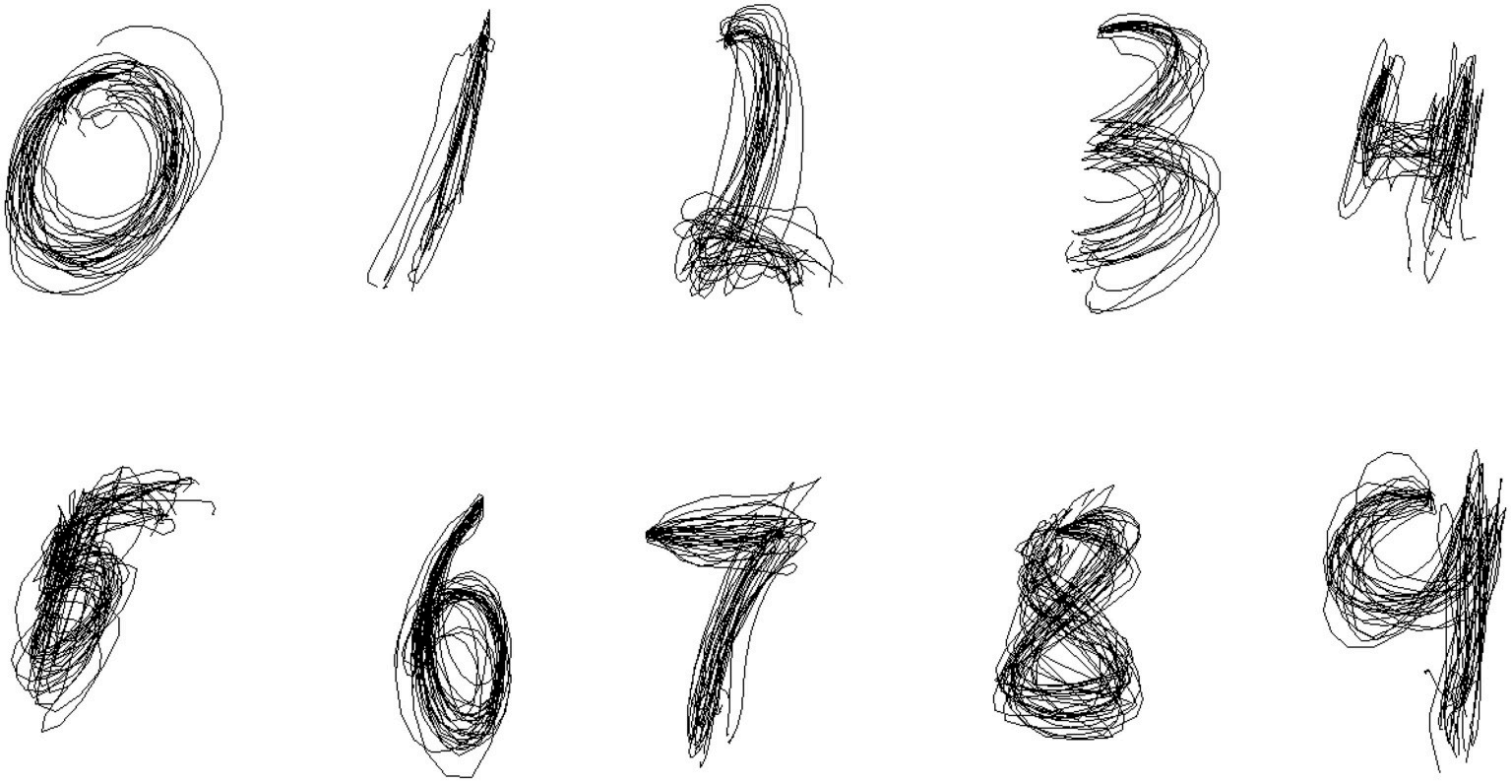
**Between-Group Reconstruction: several reconstructed trials of each symbol from cross-validation sample of one of the participants**.

In the Between-group design, we attempted to learn the parameters of the Kalman Filter for each symbol separately, and then reconstruct the traces of the same symbol. Figure 6 shows the results of reconstruction of several trials of each symbol for one of the participants. As predicted, the separate Kalman Filters for each symbol perform a more specific and accurate reconstruction, which is visually evident from the figure.

The average reconstruction in the two coordinates between subjects was 78 ± 13% and 88 ± 7%, respectively. Table 2 reports 95% confidence intervals for the average reconstruction accuracy of each symbol with separate Kalman Filters.

**Table 2:** **Between-Group Reconstruction Performance: 95% confidence intervals for the average reconstruction accuracy of each symbol between the 6 participants**.

	**Average performance, *R*^2^**		
**Symbol**	**X-coordinate**	**Y-coordinate**	**Average: (X**+**Y)/2**
“0”	0.84 ± 0.06	0.86 ± 0.05	0.85 ± 0.05
“1”	0.63 ± 0.15	0.97 ± 0.01	0.80 ± 0.08
“2”	0.82 ± 0.11	0.92 ± 0.06	0.87 ± 0.09
“3”	0.75 ± 0.12	0.93 ± 0.06	0.84 ± 0.08
“4”	0.81 ± 0.11	0.81 ± 0.03	0.81 ± 0.05
“5”	0.80 ± 0.10	0.86 ± 0.06	0.83 ± 0.07
“6”	0.83 ± 0.05	0.88 ± 0.05	0.85 ± 0.05
“7”	0.76 ± 0.20	0.88 ± 0.08	0.82 ± 0.14
“8”	0.76 ± 0.12	0.86 ± 0.07	0.81 ± 0.09
“9”	0.81 ± 0.09	0.84 ± 0.08	0.83 ± 0.08
All	0.78 ± 0.13	0.88 ± 0.07	0.83 ± 0.08

### 3.4. Comparison with the Wiener Filter

In the previous subsection we have shown that, as expected, the Kalman Filter improves the reconstruction performance, as compared to the previously proposed method (Linderman et al., 2009). The parameter search (Section 3.1) shows that adding non-zero autoregressive lag(s) to the model, and as a result, capturing the dynamical properties of the system, leads to the increase in accuracy of reconstruction for all subjects (Figures 3, 4).

To consider the increase in accuracy, specifically associated with the dynamical model, we reconstructed pen traces of several symbols by Within-group design (one set of parameters for all symbols), and then repeated the procedure on the same samples using the Wiener Filter, fixing all other external parameters, including those related to the training-testing split of the data and data preprocessing techniques.

**Figure 7:**
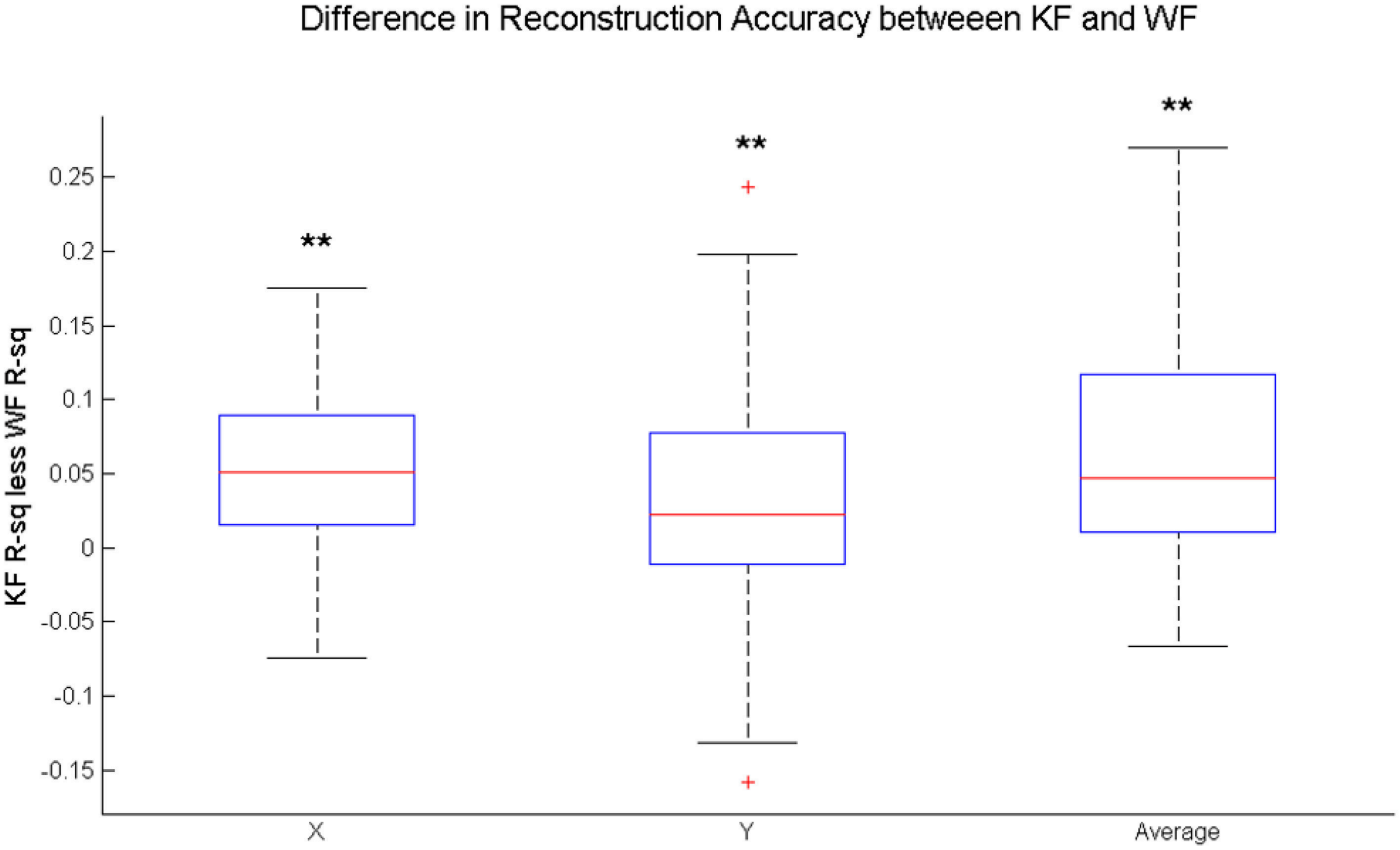
**Distribution of the difference in the average performance of the Kalman Filter (KF) and the average performance of the Wiener Filter (WF) for all written characters across subjects**. Statistical Difference between the average KF and the average WF performance is measured by one-sided Wilcoxon matched-pair test for X-coordinate, Y-coordinate and the average of the two, i.e., (X+Y)/2: ^**^significantly greater than zero at 1% significance level.

**Figure 8:**
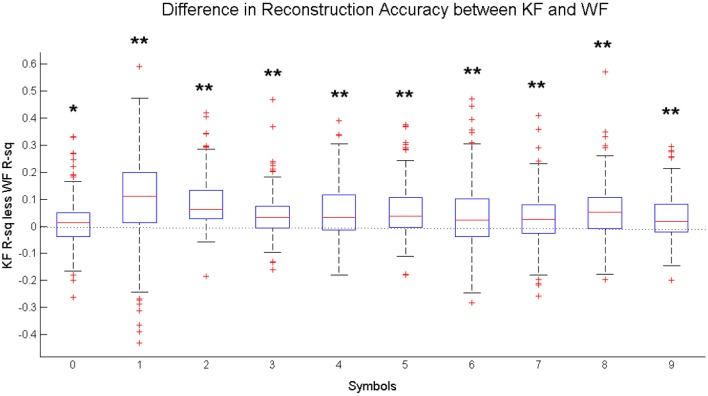
**Distribution of the difference between the Kalman Filter Accuracy (KF *R*^2^) and the Wiener Filter Accuracy (WF *R*^2^) between subjects, measured for each written character independently**. Statistical Difference between the KF and the WF performance is measured by one-sided Wilcoxon matched-pair test for each character: ^*^significantly greater than zero at 5% significance level, ^**^significantly greater than zero at 1% significance level.

On average, introduction of the KF framework leads to a significant increase in accuracy for both reconstructed coordinates across the six participants (Figure 7). The increase is more or less homogeneous between different symbols. Figure 8 shows the distribution of the difference between the Kalman Filter accuracy and the Wiener Filter accuracy for separate symbols. For all symbols, the difference is significantly greater than zero with at least 95% confidence, guaranteed by the one-sided Wilcoxon test for matched pairs.

**Figure 9:**
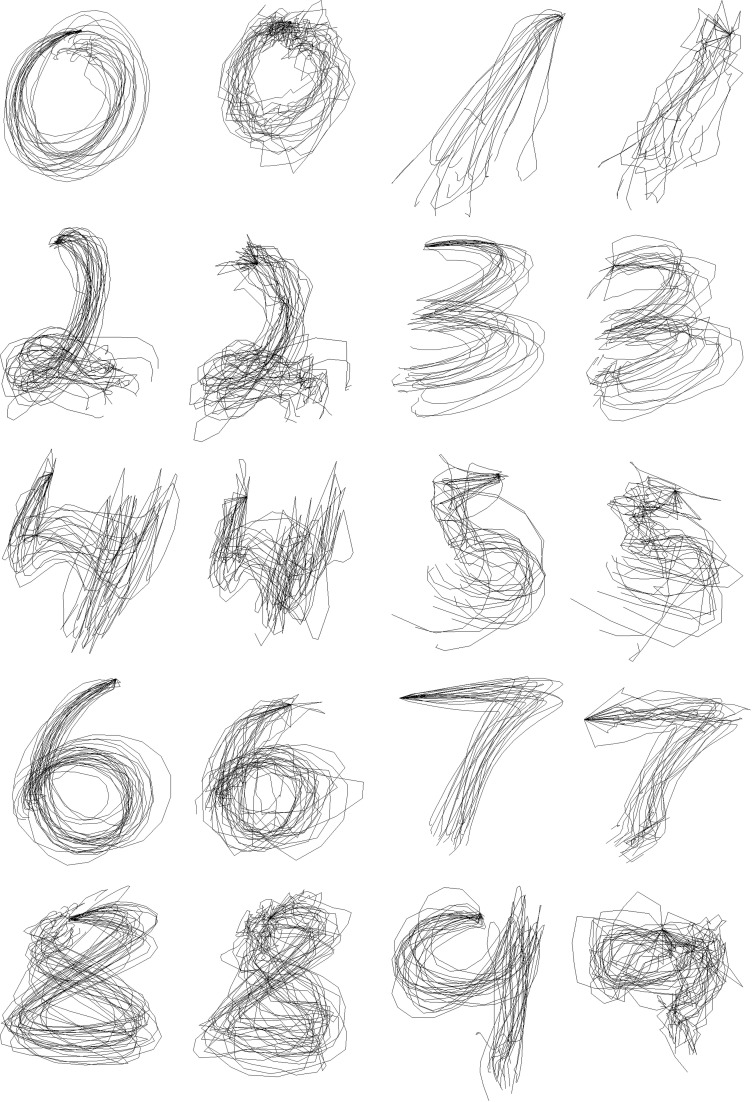
**Pen trace reconstruction of digits zero to nine with the Kalman Filter (left) and the Wiener Filter (right), Within-Group Method for several cross-validation trials of one of the participants**.

The ergonomics of the reconstructed handwriting traces plays an important role. The use of the dynamical model to enforce natural smoothness of handwriting yielded improved ergonomics of the recovered traces. Figure 9 allow to visually compare the reconstruction of the pen traces for each symbol obtained with the two methods (left—the Kalman Filter reconstruction, right—the Wiener Filter reconstruction). As we can see from the figures, the use of the Kalman filter furnishes a smoother coordinate reconstruction than that based exclusively on the measurements. While both filters make it possible to visually discriminate between different symbols, the smoothness of the traces, obtained with the Kalman filter, makes them more natural and ergonomically plausible.

## 4. Discussion

In this work we applied the Kalman Filter approach to reconstruction of handwritten pen traces on the basis of EMG measurements. We have demonstrated that it is possible to obtain accurate and ergonomic reconstruction of pen traces and still remain in the linear framework to utilize all the benefits associated with it. Our results show a significant improvement over the figures, previously reported by Linderman et al. (2009). In contrast to that pioneering work, we used only causal filtering and exploited fewer past samples of the EMG signals.

Although the accuracy of reconstruction does not generally go above 90%, in terms of the coefficient of determination, the method still offers a reliable and ergonomic reconstruction for all symbols. Approximately 25 trials of each symbol were enough to learn the parameters of the filter and yield comparable results in testing samples.

The method works well for both specific (Between-Group design) and general (Within-Group design) models, which reveals its potential for a wide range of applications. The Between-group design, although quite limited at first sight, is not entirely unrealistic, due to the fact that handwritten figures are very well discriminated between each other on the basis of EMG signals (Linderman et al., 2009; Huang et al., 2010). It means that separate Kalman filters can be applied as a second-stage algorithm in the off-line experiments, after another algorithm (such as the Hidden Markov Model of Linear Discriminant Analysis) is used to classify the symbols into groups.

The Within-group design, on the other hand, is more applicable to on-line handwriting reconstruction, when no prior information about the class of the symbol being written is available. In this framework the causality of our approach offers additional benefits and makes the EMG-controlled handwriting feel natural to the user. Also, the natural smoothness of the traces, recovered with the use of the KF, provides for an improved feedback, which is crucial in the real-life on-line scenario.

While our method has shown improvement over the previously proposed technique, it still requires thorough consideration before it can be reliably applied in neuroprosthetic devices and rehabilitation practices. One of the main difficulties in applying the Linear Kalman filter is the high variability of results across subjects. The confidence intervals in Tables 1, 2 clearly show very high margins of error, which indicate that handwriting is very person-specific.

Such heterogeneous performance across individuals apparently stems from a combination of behavioral and physiological factors, which we could not control in this study. Participants vary in the style and neatness of handwriting, including the way they hold and press the pen, and the strategies they apply to write the same symbol. Anatomical differences, such as the individual muscle length, muscle size and attachment to the bones and the differences in the amount of subcutaneous fat might be significant factors influencing the patterns of the recorded neuromuscular activity (EMGs). Additional source of variability may come from the variation in electrode placement sites.

The problem of EMG variability has received significant attention in the recent experimental literature (Linssen et al., 1993; Araújo et al., 2000; Nordander et al., 2003). Fundamentally, one and the same movement can be reproduced by different force patterns in multiple agonist and antagonist muscles. This phenomenon, called *motor redundancy* (Bernstein, 1967; Guigon et al., 2008) allows a certain kinematic pattern to be reproduced by virtually infinite number of distinct muscular activation patterns (Amis et al., 1979). The EMG recordings which we used captured relatively consistent EMG patterns in individual subjects, which were, however, different from subject to subject. The approach proposed in this paper appears to have sufficient generalization power to capture the within subject variability (both natural and the one that stems from instructed variations of symbol writings to be produced). However, initial training of the algorithm is required for each individual subject independently.

Additionally, the results can be further improved by individually tuning the latent parameters, such as the model orders, filter cut-off frequency and sensor locations. In this work, however, we intentionally used a single set of latent variable values in order to emulate the out-of-box performance of such a system. Methods for non-supervised on-line adaptation and individual tuning of the latent variables need to be developed to address these issues and make the fine-tuning seamless to the user.

The linear framework of the filter offers significant benefits, such as stability and good generalization ability. However, the non-linear nature of the relation between the recorded EMG signals and the actuator trajectory prompts to explore the use of non-linear models in this application. The benefits brought in by the non-linearity, however, have to be leveraged against the additional complexity and potential instability associated with the use of such models.

## 5. Conclusions

In this paper we investigated the relationship between handwriting and neuromuscular activity measured by electromyography. We built and optimized the Kalman filter in order to reconstruct the pen coordinates based on the dynamical characteristics of handwriting and the corresponding EMG measurements.

We showed that the Kalman filter significantly outperforms previously proposed method (Linderman et al., 2009) and yields a mean accuracy of 68% in Within-Group design and 83% in Between-Group design, measured by the coefficient of determination, averaged for the two reconstructed coordinates. Our method is suitable for real-time applications as it is causal and utilizes only the EMGs from the past. The dynamical nature of the Kalman filter provides for the time-varying optimal fusion of the information and allows to take into account not only the EMG activity, but also the physical properties of handwriting.

The main attraction of the proposed method is its ability to smooth the noise and, as a result, provide a comprehensible and realistic reconstruction. Further progress in this field would potentially create intelligent rehabilitation techniques for patients with hand injuries, as well as become useful in human-computer interfaces, associated with handwriting.

### Conflict of interest statement

The authors declare that the research was conducted in the absence of any commercial or financial relationships that could be construed as a potential conflict of interest.

## Appendix

### Parameters of the state transition model

Equation (A3) shows the distribution of the estimate of the State Transition Model given by Equation (1).

The mean of the distribution is

(A1) $$\mu_{1t}=E[s_{t}]=A{\hat{s}}_{t-1}=A\mu_{1(t-1)}.$$

The covariance matrix is derived the following way:

$$\begin{array}{l} \;\Sigma_{1t}=E[(s_{t}-\mu_{1t}){\left(s_{t}-\mu_{1t}\right)}^{T}]=\\ \;=E[(As_{t-1}+v_{t}-A{\hat{s}}_{t-1}){\left(As_{t-1}+v_{t}-A{\hat{s}}_{t-1}\right)}^{T}]=\\ \;=E[(A(s_{t-1}-{\hat{s}}_{t-1})+v_{t}){\left(A\left(s_{t-1}-{\hat{s}}_{t-1}\right)+v_{t}\right)}^{T}]=\\ \;=E[(A(s_{t-1}-{\hat{s}}_{t-1})+v_{t})({\left(s_{t-1}-{\hat{s}}_{t-1}\right)}^{T}A^{T}+v_{t}^{T})]=\\ =AE[(s_{t-1}-{\hat{s}}_{t-1}){\left(s_{t-1}-{\hat{s}}_{t-1}\right)}^{T}]A^{T}+AE[(s_{t-1}-{\hat{s}}_{t-1})v_{t}^{T}]\\ \;+E[v_{t}{\left(s_{t-1}-{\hat{s}}_{t-1}\right)}^{T}]A^{T}+E[v_{t}v_{t}^{T}]. \end{array}$$

Since the state estimate noise ***v***_*t*_ is uncorrelated with the estimate,

$$E[v_{t}{\left(s_{t-1}-{\hat{s}}_{t-1}\right)}^{T}]=E[(s_{t-1}-{\hat{s}}_{t-1})v_{t}^{T}]=0$$

and the covariance matrix reduces to:

(A2) $$\begin{array}{l} \Sigma_{1t}=AE[(s_{t-1}-{\hat{s}}_{t-1}){\left(s_{t-1}-{\hat{s}}_{t-1}\right)}^{T}]A^{T}+E[v_{t}v_{t}^{T}]=\\ \;=A\Sigma_{1(t-1)}A^{T}+Q. \end{array}$$

### The fusion

Under the normality assumption the probability density function (pdf) of the state space vector ***s***_*t*_ can be written as

(A3) $$\begin{array}{l} f_{1}(s_{t}|s_{t-1})={\left(2\pi \right)}^{-3K}|\Sigma_{1t}{|}^{-1/2}\exp(-\frac{1}{2}{\left(s_{t}-\mu_{1t}\right)}^{T}\\ \;\Sigma_{1t}^{-1}(s_{t}-\mu_{1t})) \end{array}$$

Since the additive noise term ***w***_*t*_ in Equation (2) is assumed to be Gaussian, while each of the observed EMG signals are not modeled as stochastic processes, the state vector follows the Gaussian distribution

(A4) $$f_{2}(s_{t}|z_{t})={\left(2\pi \right)}^{-4L}|\Sigma_{2t}{|}^{-1/2}\exp(-\frac{1}{2}{\left(s_{t}-\mu_{2t}\right)}^{T}\Sigma_{2t}^{-1}(s_{t}-\mu_{2t})).$$

By multiplying pdf in Equation (A3) by pdf in Equation (A4) we get a scaled fused Distribution of the state estimate:

(A5) $$\begin{array}{l} \;f_{fused}(s_{t}|s_{t-1},z_{t})=f_{1}(s_{t}|s_{t-1})f_{2}(s_{t}|z_{t})=\\ N(\mu_{1t},\Sigma_{1t})\;\cdot \;N(\mu_{2t},\Sigma_{2t})=cN(\mu_{fused},\Sigma_{fused}), \end{array}$$

with parameters

(A6) $$\Sigma_{Fused}={\left(\Sigma_{1t}^{-1}+\Sigma_{2t}^{-1}\right)}^{-1}$$

(A7) $$\mu_{Fused}=\Sigma_{Fused}(\Sigma_{1t}^{-1}\mu_{1t}+\Sigma_{2t}^{-1}\mu_{2t})$$

and a normalization constant

(A8) $$\begin{array}{l} c=\frac{1}{{\left(2\pi \right)}^{k/2}}\frac{|\Sigma_{Fused}{|}^{1/2}}{|\Sigma_{1t}{|}^{1/2}|\Sigma_{2t}{|}^{1/2}}\exp(-\frac{1}{2}(\mu_{1t}^{T}\Sigma_{1t}^{-1}\mu_{1t}\\ \;+\mu_{2t}^{T}\Sigma_{2t}^{-1}\mu_{2t}+\mu_{Fused}^{T}\Sigma_{Fused}^{-1}\mu_{Fused})) \end{array}$$

Let us reformulate the expressions for the mean and Covariance matrix of the new distribution in such a way that they separate the influence of the two fused distributions.

Using the Matrix Inversion lemma (the Woodbury matrix identity) for two matrices of the same dimension (**Σ**_1*t*_ and **Σ**_2*t*_) and setting $K_{t}=\Sigma_{1t}{\left(\Sigma_{1t}+\Sigma_{2t}\right)}^{-1}$ the parameters of the fused Gaussian can be neatly rewritten in the form of Equations (8) and (9), i.e.,

(A9) $$\Sigma_{Fused}=\Sigma_{1t}-\Sigma_{1t}{\left(\Sigma_{1t}+\Sigma_{2t}\right)}^{-1}\Sigma_{1t}=\Sigma_{1t}-K_{t}\Sigma_{1t}.$$

(A10) $$\begin{array}{l} \;\mu_{Fused}=(\Sigma_{1t}-K_{t}\Sigma_{1t})(\Sigma_{1t}^{-1}\mu_{1t}+\Sigma_{2t}^{-1}\mu_{2t})=\\ \;=\mu_{1t}+\Sigma_{1t}\Sigma_{2t}^{-1}\mu_{2t}-K_{t}\mu_{1}-K_{t}\Sigma_{1t}\Sigma_{2t}\mu_{2t}=\\ \;=\mu_{1t}+K_{t}(K_{t}^{-1}\Sigma_{1t}\Sigma_{2t}^{-1}\mu_{2t}-\mu_{1t}-\Sigma_{1t}\Sigma_{2t}^{-1}\mu_{2t})=\\ =\mu_{1t}+K_{t}((\Sigma_{1t}+\Sigma_{2t})\Sigma_{1t}^{-1}\Sigma_{1t}\Sigma_{2t}^{-1}\mu_{2t}-\mu_{1t}-\Sigma_{1t}\Sigma_{2t}^{-1}\mu_{2t})=\\ \;=\mu_{1t}+K_{t}(\Sigma_{1t}\Sigma_{2t}^{-1}\mu_{2t}+\mu_{2t}-\mu_{1t}-\Sigma_{1t}\Sigma_{2t}^{-1}\mu_{2t})=\\ \;=\mu_{1t}+K_{t}(\mu_{2t}-\mu_{1t})=(I-K_{t})\mu_{1t}+K_{t}\mu_{2t}. \end{array}$$

### Testing the assumptions of the model

We ran the Royston test for multivariate normality on the residuals of each training sample and concluded that for most samples the assumption of normality is not violated (at 1% significance level). Table A1 summarized the percentages of samples, in which the assumption of normality of the error term holds in the two reconstructed models and in the joint model.

**Table A1:** **Percentages of trials across participants, in which the assumption of multivariate normality of the error term is not violated at 1% significance level**.

	**Participants**						
	**1 (%)**	**2 (%)**	**3 (%)**	**4 (%)**	**5 (%)**	**6 (%)**	**Average (%)**
State transition model	92	79	92	96	83	73	86
Measurement model	93	85	92	94	80	77	87
Joint model	89	72	89	93	76	67	81

The fusion of the two information sources described in this paper is heavily based on the assumption that the two merged sources are independent.

Then, we checked to what extent the two information sources can be considered independent. Taking into account the results of the test for multivariate normality, we checked for the absence of correlation between the error terms of the two merged models (Equations 1 and 2). We used the residuals of the two models to calculate the sample cross-correlation matrix and assessed the significance of its elements for each trial.

Given the sample size and the multiplicity of the tested hypothesis (36), we could not reject the null-hypothesis of no-correlation at 5% group level significance for the majority of trials (74%). Detailed distribution of the test performance across the participants is given in Table A2.

**Table A2:** **Percentages of trials across participants, in which the test for uncorrelatedness of residuals of the two fused models was not rejected at 5% significance level**.

	**Participants**						
	**1 (%)**	**2 (%)**	**3 (%)**	**4 (%)**	**5 (%)**	**6 (%)**	**Average (%)**
Independent trials	83	72	76	80	67	68	74
