# Corrigendum: A dynamical model improves reconstruction of handwriting from multichannel electromyographic recordings

**DOI:** 10.3389/fnins.2015.00517

**Published:** 2016-01-25

**Authors:** Elizaveta Okorokova, Mikhail Lebedev, Michael Lindermann, Alex Ossadtchi

**Affiliations:** ^1^Centre for Cognition and Decision Making, National Research University Higher School of EconomicsMoscow, Russia; ^2^Department of Neurobiology, Duke UniversityDurham, NC, USA; ^3^Department of Biomedical Engineering, Norconnect Inc.Ogdensburg, NY, USA; ^4^Laboratory of Control of Complex Systems, Institute of Problems of Mechanical Engineering, Russian Academy of SciencesSt. Petersburg, Russia

**Keywords:** handwriting, electromyography, pattern recognition, dynamical modeling, the Kalman filter, the Wiener filter

The correct acknowledgment should read:

The authors are grateful for the fruitful comments of Dr. Igor Furtat related to the algorithmic portion of the work. The experimental portion (Section 3) was prepared within the framework of a subsidy granted to the National Research University Higher School of Economics by the Government of the Russian Federation for the implementation of the Global Competitiveness Program. The data fusion algorithm for reconstruction of pen traces (Section 2) was adapted to the use with the EMG data solely under support of RNF (grant 14-29-00142) in IPME RAS.

The change requested does not affect the scientific validity of the results.

## Conflict of interest statement

The authors declare that the research was conducted in the absence of any commercial or financial relationships that could be construed as a potential conflict of interest.

